# Keeping pace with forestry: Multi-scale conservation in a changing production forest matrix

**DOI:** 10.1007/s13280-019-01248-0

**Published:** 2019-09-16

**Authors:** Adam Felton, Therese Löfroth, Per Angelstam, Lena Gustafsson, Joakim Hjältén, Annika M. Felton, Per Simonsson, Anders Dahlberg, Matts Lindbladh, Johan Svensson, Urban Nilsson, Isak Lodin, P. O. Hedwall, Anna Sténs, Tomas Lämås, Jörg Brunet, Christer Kalén, Bengt Kriström, Pelle Gemmel, Thomas Ranius

**Affiliations:** 1 Southern Swedish Forest Research Centre, SLU, Box 49, Rörsjöv 1, 230 53 Alnarp, Sweden; 2 Department of Wildlife, Fish, and Environmental Studies, Swedish University of Agricultural Sciences, 901 83 Umeå, Sweden; 3 Faculty of Forest Sciences, School for Forest Management, Swedish University of Agricultural Sciences, PO Box 43, 730 91 Skinnskatteberg, Sweden; 4 Department of Ecology, Swedish University of Agricultural Sciences, P.O. Box 7044, 750 07 Uppsala, Sweden; 5 Härnösand, Sweden; 6 Department of Forest Mycology and Plant Pathology, Swedish University of Agricultural Sciences, PO Box 7026, 750 07 Uppsala, Sweden; 7 Department of Historical, Philosophical and Religious Studies, Umeå University, 901 87 Umeå, Sweden; 8 Department of Forest Resource Management, Swedish University of Agricultural Sciences, 901 83 Umeå, Sweden; 9 National Forest Agency, Bryggargatan 19-21, 503 38 Borås, Sweden; 10 Department of Forest Economics, Swedish University of Agricultural Sciences, 90183 Umeå, Sweden

**Keywords:** Biodiversity conservation, Climate change mitigation, Even-aged forestry, Green-tree retention, Habitat loss, Protected areas

## Abstract

The multi-scale approach to conserving forest biodiversity has been used in Sweden since the 1980s, a period defined by increased reserve area and conservation actions within production forests. However, two thousand forest-associated species remain on Sweden’s red-list, and Sweden’s 2020 goals for sustainable forests are not being met. We argue that ongoing changes in the production forest matrix require more consideration, and that multi-scale conservation must be adapted to, and integrated with, production forest development. To make this case, we summarize trends in habitat provision by Sweden’s protected and production forests, and the variety of ways silviculture can affect biodiversity. We discuss how different forestry trajectories affect the type and extent of conservation approaches needed to secure biodiversity, and suggest leverage points for aiding the adoption of diversified silviculture. Sweden’s long-term experience with multi-scale conservation and intensive forestry provides insights for other countries trying to conserve species within production landscapes.

## Introduction

The ongoing global loss of species and ecosystems (Ceballos et al. 2015; IPBES 2019), and the demonstrated importance of biodiversity to human well-being (MEA 2005; Cardinale et al. 2012), is driving national and international efforts to conserve biodiversity (CBD 2010). Conserving sufficient amounts of the world’s varied forest ecosystems is critical, due to the biodiversity and ecosystem services these systems provide (Brockerhoff et al. 2017). Because natural forest ecosystems exhibit structures and dynamics that are highly variable in space and time (Angelstam 1998; Kuuluvainen 2009), conserving forest biodiversity requires the maintenance, and often restoration, of forest habitat over multiple scales (Lindenmayer and Fischer 2006). However, a large part of the world’s forests are managed for wood production and other economic, environmental, or cultural values, and only 13% of the world’s forests are formally protected for biodiversity conservation (FAO 2016). Thus, effective forest biodiversity conservation must rely on habitat contributions from both protected forests and forests actively managed for the production of biomass and other goods and services. In many regions, these production forest lands form the ‘matrix’, which is the most extensive land-use and vegetation category, and thus has a dominant influence on ecological processes at the landscape scale (Forman 2014). Depending on the focal species, this matrix can provide suitable habitat, or the ecological context within which suitable habitat is located (Lindenmayer and Fischer 2006; Forman 2014).

Multi-scale conservation is an approach used to conserve biodiversity in such forest landscapes (Lindenmayer et al. 2006). Typically this approach combines landscape-scale protected forest areas, intermediate-scale reserves set within the production forest matrix, and at the smallest scale, the retention of key habitat features (e.g. buffer zones, old large trees, dead wood) within production stands (Lindenmayer et al. 2006; Simonsson 2016). Although the specifics vary, multi-scale conservation is applied on several continents, from the temperate forests of Tasmania, South America and the Pacific NW of USA, to the boreal forests of Northern Europe and Canada (Gustafsson and Perhans 2010; McDermott et al. 2010). A central premise is that since species vary in the spatial scale of their habitat requirements, and capacity to persist in non-protected areas, when used in combination protected and non-protected areas should more efficiently sustain viable populations of species (Lindenmayer and Franklin 2002). Achieving this outcome is however complicated, as it requires balancing the representativity, amount and connectivity of protected forest areas, with ongoing changes in land-use intensity and habitat provision in the production forest matrix.

In many regions, past land-use has limited the possibility of relying on remaining large, contiguous, and high value protected areas for biodiversity conservation (Branquart et al. 2008). Globally, 50% of remaining intact forests are within 500 m of forest edges, and most intact forest fragments are 10 ha or less (Haddad et al. 2015). Under such circumstances, the intensity of production forest management becomes important for forest biodiversity conservation. The intensity of forestry practice refers to the extent natural forest development is altered to enhance production (see Duncker et al. 2012). Intensive forestry generally results in a greater divergence of stand variables and parameters (e.g. tree species composition, disturbance regimes, forest structures) from natural forest conditions and native species’ habitat requirements (Felton et al. 2016a). Current trends indicate that global reliance on intensively managed production forests (e.g. planted forests, even-aged forestry) will continue to increase (Warman 2014; Payn et al. 2015) due to economic incentives (Puettmann et al. 2015), growing advocacy for the “bioeconomy” (Winkel 2017), and the need to mitigate climate change (Williamson 2016). In opposition to these trends, there is growing international awareness of the potential biodiversity and ecosystem service benefits from diversifying silviculture to include a wider variety of less intensive practices (Puettmann et al. 2015) that better match natural forest disturbance regimes and tree species composition (Angelstam 1998; Kuuluvainen 2009). Less intensive silvicultural practices can provide greater forest structural complexity and small-scale variability than even-aged approaches (uneven-aged forestry; Kuuluvainen et al. 2012a, b), and a higher diversity of tree species (mixed-species stands; Pretzsch et al. 2017), with associated benefits for forest biodiversity (Lindenmayer and Franklin 2002).

Since the late 1980s, Sweden has been applying a multi-scale approach to forest biodiversity conservation (Gustafsson and Perhans 2010). Under this framework, most of Sweden’s productive forest area (i.e. capable of producing ≥ 1 m^3^ of wood ha^−1^ yr^−1^) continues to be managed intensively using even-aged approaches for the production of timber, pulp and bioenergy. Within this production forest matrix, Sweden has increased both the spatial extent of protected forest areas and voluntary set-asides (Angelstam et al. 2011; Elbakidze et al. 2013). Furthermore, the integration of conservation considerations within production forest (e.g. green-tree retention) has also increased (SFA 2014). Nevertheless, semi-natural forest remnants continue to be harvested and fragmented (Svensson et al. 2018; Jonsson et al. 2019), and over 2000 forest-associated species (of 15 000 assessed) are listed as threatened on Sweden’s red-list, largely represented by macro-fungi, beetles, lichens and butterflies (Sandström 2015). Many red-listed species are threatened specifically by forest felling (Sandström 2015). Recent evaluations concluded that Sweden is not on track to meet its own national 2020 environmental goals for sustainable forests (SEPA 2018).

As an early adopter of multi-scale conservation, and one of the world’s leading producers of forest products (SFIF 2018), Sweden’s experiences provide internationally relevant insights regarding the opportunities and obstacles for other countries trying to successfully integrate multi-scale conservation efforts with forest production. Although the full consequences of these efforts are not yet seen, sufficient time has passed to consider whether current trajectories appear on track with defined targets for conserving Sweden’s forest biodiversity. Here we use these circumstances to highlight the importance of the production forest matrix and its management for the success of multi-scale conservation. To address these issues, we summarize trends in habitat provision by Sweden’s protected and production forest areas, and overview the diverse ways in which production forestry can intensify or diversify. We then discuss the potential implications of intensified versus diversified production forest trajectories for the amount and type of conservation interventions needed, and discuss the implications of these trajectories for increasing habitat availability and better securing the status of forest biodiversity. By so doing, we identify several key knowledge gaps whose resolution is relevant to the success of multi-scale conservation in Sweden and elsewhere, and identify several leverage points for aiding the adoption of more diversified forestry practices.

## Sweden’s forest circumstance

Forests cover 70% of Sweden’s land area (comprising both temperate and boreal biomes), and the majority of productive forest area is used for forestry. Despite only being the world’s 55th largest country, Sweden has the fifth largest total planted forest area (Payn et al. 2015), and has one of the highest wood extraction intensities (harvested volume to annual increment) in Europe (Levers et al. 2014). This enables Sweden with just 1% of the world’s productive forest land to be the third largest exporter of pulp, paper and sawn timber (SFIF 2018). Sweden achieves this almost exclusively via even-aged silviculture, efficient harvesting systems, the extensive use of planted seedlings (SFA 2018c), and two native conifer species, Norway spruce (*Picea abies*) and Scots pine (*Pinus sylvestris*), which comprise 80% of standing volume (SFA 2014). In terms of control, small-scale private ownership shows a clear latitudinal gradient from 76% in the south to 36% in the north, where state and private forestry companies dominate (SFA 2014).

Concerns regarding the impacts of intensive forestry on forest biodiversity resulted in two key amendments to the Swedish Forestry Act in the early 1990s: the provision of equal status to environmental and production objectives, and the deregulation of forestry from a previously centralized prescriptive system (Gov. bill 1992/93:226, 58; Lämås and Fries 1995; Bush 2010). As a result, Sweden’s forest governance model has few prescriptive stipulations (Lindahl et al. 2017), and instead relies on soft policy instruments such as information, advice and education (Appelstrand 2012). To achieve equity between production and environmental objectives, more forest area was set aside for conservation, and environmental considerations increased within production forests (e.g. green-tree retention at harvest). Deregulation was expected to increase the diversity of production forest management practices, and thereby further benefit biodiversity (Lämås and Fries 1995; Bush 2010; Stens et al. in press). Voluntary certification schemes (i.e. Forest Stewardship Council, FSC; Programme for the Endorsement of Forest Certification, PEFC), and forest biodiversity education campaigns, were used widely to support forest policy implementation (Gustafsson and Perhans 2010; Johansson et al. 2013). The official target for biodiversity is that “Species habitats and ecosystems and their functions and processes must be safeguarded” and “species must be able to survive long-term in viable populations with sufficient genetic variation” (Regeringskansliet and Miljödepartementet 2012).

## Developments in protected and production forest habitat

**Fig. 1:**
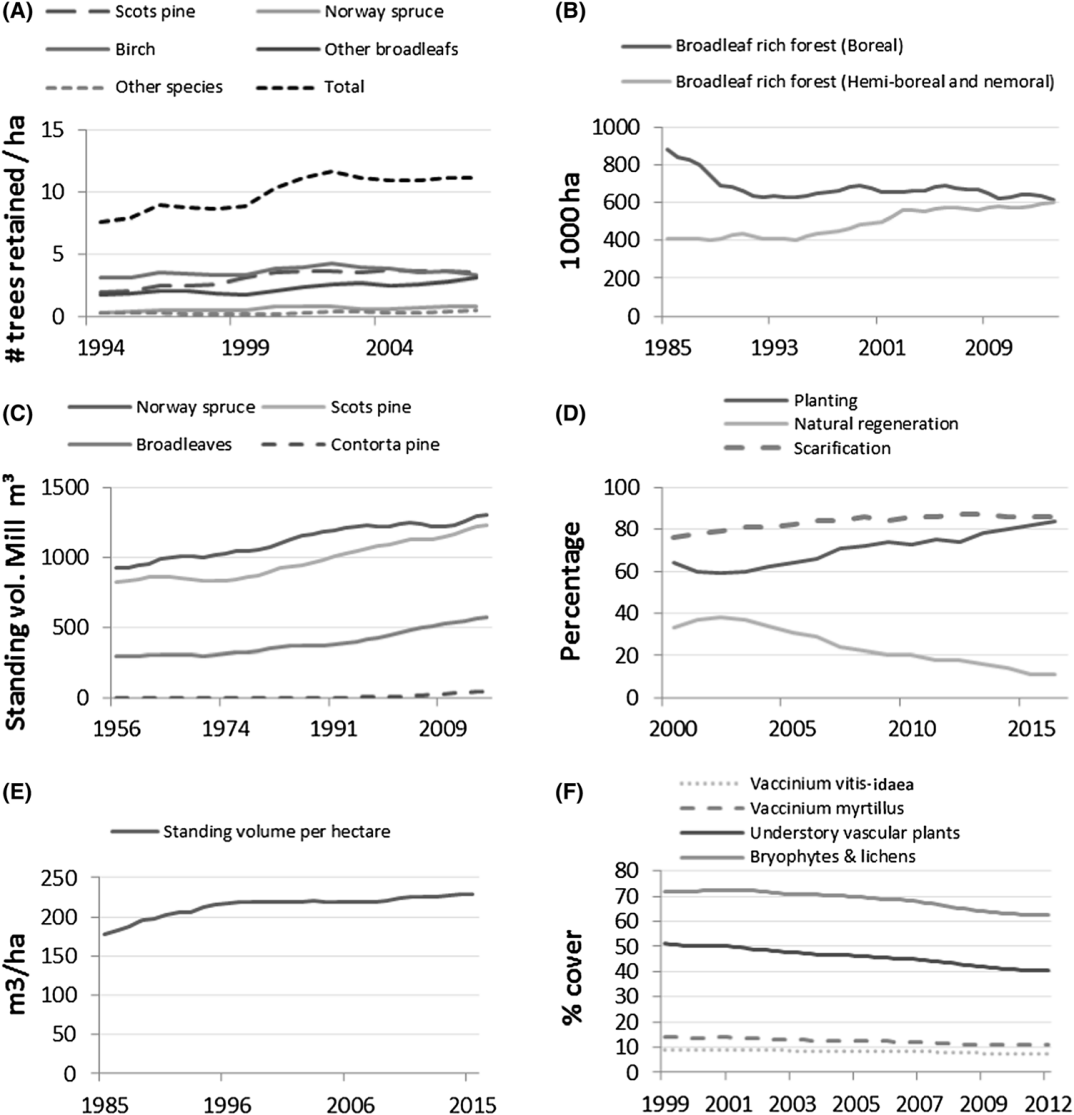
National trends in forest variables as collected by the Swedish National Forest Inventory (SEPA 2018; SFA 2018c; SLU 2018). **a** Trees with diameter > 15 cm retained after final felling, as surveyed 5–7 years later. **b** Area covered by boreal forest over 80 years of age, and hemi-boreal and temperate forest over 60 years of age that have a basal area of at least 25% broadleaved trees. **c** Standing volume for select tree species and classes in millions of cubic metre on productive forest land. **d** Regeneration method and use of scarification as a percentage of logged area. **e** Standing volume per hectare at the age of final felling. **f** Percentage cover of ground layer vegetation, specifically cowberry *Vaccinium vitis*-*idaea*, bilberry *Vaccinium myrtillus*, all vascular plants and all bryophytes and lichens, on production forest land. Analyses exclude protected areas as of 2015 (**b**, **e**) or 2017 (**c**, **f**). The time period provided differs depending on data availability

Since the 1994 Forestry Act, Sweden has substantially increased the amount of formally protected forest area from 0.5% (Statistics Sweden 1994) to just over 4% of productive forest land (SLU 2018), although the largest protected areas are limited to the northwestern less productive and less species-rich forests (Gustafsson et al. 2015). For perspective, the proportion of total forest area formally protected globally today is 13% (FAO 2016). Five percent of productive forest land in Sweden is also voluntarily set aside from production (SFA 2014). Voluntary set-asides range from 0.5 to 20 ha and complement formally protected areas (Simonsson 2016), though they do not legally ensure long-term protection, nor that the most valuable forest habitats for biodiversity are prioritized (Michanek et al. 2018). In addition, 14% of total forest area consists of unproductive forest (< 1 m^3^ ha^−1^ year^−1^) that is neither protected (e.g. national park, reserve, conservation agreement) nor available for commercial forestry (SFA 2014). At the smallest scale of conservation, certification requires that individual trees or groups of trees of higher conservation value (FSC certification requirements stipulate 10 per ha; ideally larger/older broadleaves) are excluded from harvesting at clear felling (Johansson et al. 2013). Additional certification requirements stipulate the creation of high stumps, retention of certain categories of dead trees, special provisions for broadleaf trees, the use of buffer zones, and the protection of sensitive habitats from logging operations (FSC 2010). One recent assessment estimates that such retained patches of forest represent approximately 11% of harvested areas one year after final felling (Skogsstyrelsen 2019). The number of retained trees on harvested areas have also increased in recent years (Fig. 1a), as have dead wood levels from 6 m^3^ to 8 m^3^ ha^−1^ since 1996 (SLU 2016). Since the 1994 Forestry Act, the area of ‘old’ (> 120 years temperate/hemi-boreal, > 140 years boreal) productive forest has almost doubled (SEPA 2018), and the total area of mature broadleaf rich forest (> 30% broadleaf, older than 60 years) has also increased before stabilizing during the last 10 years (Fig. 1b, SEPA 2018).

Some positive developments have also occurred specifically in relation to production forests. For example, the area of young regenerating birch (*Betula* spp.) forest has increased from 2 to 4% in the last 20 years (NFI unpublished data, non-protected forest), likely resulting in part from the 1993 allowance to count birch as production stems when meeting regeneration requirements (Bergquist et al. 2016). Likewise, the FSC’s requirement that at least 5% (10% in the temperate and hemi-boreal region) of stand volume consists of broadleaf trees at the time of final felling is contributing to the structural and compositional diversity of production stands (FSC 2010). The proportion of young regenerating forest area (2–12 years of age) consisting of broadleaf mixtures has also increased from 3 to 5% in recent years (NFI unpublished data, non-protected forest). Although the introduced lodgepole pine (*Pinus contorta*) is the sixth most common tree species by volume (SLU 2018), and continues to increase (Fig. 1c, Bergquist et al. 2016), introduced tree species nevertheless remain a small component of production forest area in Sweden (3%, FAO 2014) relative to many other European countries (see Felton et al. 2013; Forest Europe 2015).

Overall, the structural diversity of young production forests has increased over recent decades (Kruys et al. 2013), and together with increases to broadleaf and older forest, these changes can be expected to benefit forest biodiversity (Gustafsson et al. 2010; Johansson et al. 2013; Sandström 2015). However, 60% of Sweden’s productive forest land consists of planted even-aged forests (FAO 2014), and the proportion of harvested stands subsequently planted has increased to over 80% (Fig. 1d, SFA 2018c). Meanwhile, uneven-aged forestry (Box 2) remains a rare silvicultural outlier (Axelsson et al. 2007; Stens et al. in press). The use of mechanical soil scarification has also increased (Fig. 1d, SFA 2018c), with associated negative impacts on understory vegetation (Bergstedt et al. 2008). Furthermore, the area of Norway spruce has increased from less than 28% to almost 39% of young stands (2 to 12 years, non-protected forest areas) in just 20 years (NFI unpublished data; Box 1). Despite this, the forestry sector is expressing concerns that the proportion of regenerating broadleaves (particularly birch) is too high in southern Sweden, and should be replaced with more Norway spruce or Scots pine if biomass production levels are to be increased (SFA 2018a).

**Box 1:** Four examples of ongoing forestry intensification in Sweden that illustrate the variety of ways habitat can be negatively affected. Additional intensification pathways that could be used more in the future (SFA 2018a) include fertilization (Strengbom and Nordin 2008) and exotic trees (Felton et al. 2013)

*Clearfelling of naturally regenerated continuity forests* For over 50 years, forestry has caused extensive losses to contiguous forest areas possessing long histories of forest-cover continuity, and the few remaining areas possessing such features continue to be clear-felled (Svensson et al. 2018). In recent decades, forestry has harvested 2–4 times the area of “old forest” that was protected during the same period (SEPA 2015). These forests can support old-growth and forest interior species (Johansson et al. 2007; Hjältén et al. 2012), due to the presence of key microhabitats (e.g. tree hollows, Ranius et al. 2009; kelo trees, Santaniello et al. 2017), and the increased temporal opportunities for species colonization (Keymer et al. 2000; Nordén et al. 2014). In the inland areas of northern Sweden today, the largest intact contiguous forest areas are estimated to be 2% the size of the largest areas 40–50 years ago, and only 6% of interior forest core areas persist that are likely to possess high natural values (Svensson et al. 2018). Outside the mountain foothills of northwest Sweden, most of these forests remain unprotected and at risk of clear felling (Bovin et al. 2016), unless located on impediment where forestry is not permitted. *Reliance on few production tree species* Norway spruce and Scots pine comprise 80% of standing volume in Sweden (Fig. 1c, SLU 2018). The widespread use of conifers in forestry limits restoration opportunities for native broadleaves (Lindbladh et al. 2014). This uniformity is increasing in some regions due to the planting of Norway spruce on forest sites ecologically suited to Scots pine (SFA 2018a). This stems in part from Norway spruce’ relative unpalatability to large browsing herbivores, which favours people’s use of this tree species over those more susceptible to browsing damage, including Scots pine (SFA 2017; Bergqvist et al. 2018). As a result, Norway spruce is now the most commonly chosen tree for regenerating sites in most of southern Sweden regardless of site fertility (SFA 2018d), in a region where it already comprises ~ 50% of standing volume (SFA 2014). The extensive use of conifers and the relative increase of Norway spruce increases forest uniformity and adversely affects forest biodiversity (Petersson et al. 2019). *Increasing forest density and canopy cover* Standing volume at maturity in production stands has increased by 30% since the 1980s (Fig. 1e, SLU 2018). As timber volume and canopy cover goes up, light transmission to the forest floor decreases (Korhonen et al. 2007). This is particularly the case in Norway spruce stands due to the shade produced, especially at high densities (Hedwall et al. 2013). This has negative effects on a wide range of different species groups, including understory vegetation (Fig. 1f, Hedwall et al. 2013; SLU 2017; Hedwall et al. 2019) and pollinator communities (Hanula et al. 2015). *Logging residue extraction* Forest biomass is used as a bioenergy substitute for fossil fuels (Cintas et al. 2017), obtained by extracting logging residues (branches, tops and stumps) after thinning and final felling (Ranius et al. 2018). The SFA recently concluded this practice should be increased to replace fossil fuels (Bergquist et al. 2016). In 2017, the collection of tops and branches was planned for 38% of Sweden’s final-felled areas (SFA 2018c), whereas stump harvesting remains limited (SFA 2013). Negative effects on biodiversity can therefore be expected, particularly due to habitat loss and increased site disturbance (Andersson et al. 2017b; Ranius et al. 2018). Outtake of forest biomass can therefore conflict with conservation efforts to increase woody debris in production stands (SEPA 2018). However, impacts on red-listed taxa may be small as they have limited known reliance on these materials (de Jong and Dahlberg 2017)	

Additional caveats can be made regarding observed increases in “old” forest areas (> 140 years old according to national statistics). The tree ages included in this “old” category are only a fraction of potential tree lifespans (Kuuluvainen et al. 2002), and remain too young for many forest species dependent on habitat continuity or microhabitats associated with ancient trees (Ranius et al. 2009; Santaniello et al. 2017). Relatedly, although dead wood levels have increased in production forests, the dead wood provided only constitutes a small fraction of that found in natural forest (50–120 m^3^ ha^−1^; Siitonen 2001), and is largely comprised of smaller diameter, dead wood of Norway spruce and Scots pine (Jonsson et al. 2016). Many red-listed species rely on coarser dead wood types of other tree species (Stokland et al. 2012).

In summary, despite clear increases in the amount of protected forest area, and some positive trends in habitat indicators, conservation efforts in Sweden continue to be considered inadequate from a number of perspectives. These include (i) reviews of conservation measures versus species’ habitat requirements (Johansson et al. 2013; Jonsson et al. 2016); (ii) the continued presence of approximately 2000 forest-associated species on Sweden’s red-list (the majority of which experts consider to have decreasing and fragmented populations that are sensitive to clear felling (Sandström 2015)); and (iii) the Swedish government’s own conclusion that current measures will not achieve the sustainable forest goals, due to the inadequate protection of high biodiversity forests, forest habitat loss and fragmentation (SEPA 2018). Furthermore, several once positive trends in habitat availability ( e.g. large retention trees, dead wood amounts) have recently slowed down (Ram et al. 2017), and the conservation status of fifteen of the sixteen Natura 2000 forest habitat types in Sweden are judged as inadequate (SEPA 2015).

## Discussion

**Fig. 2:**
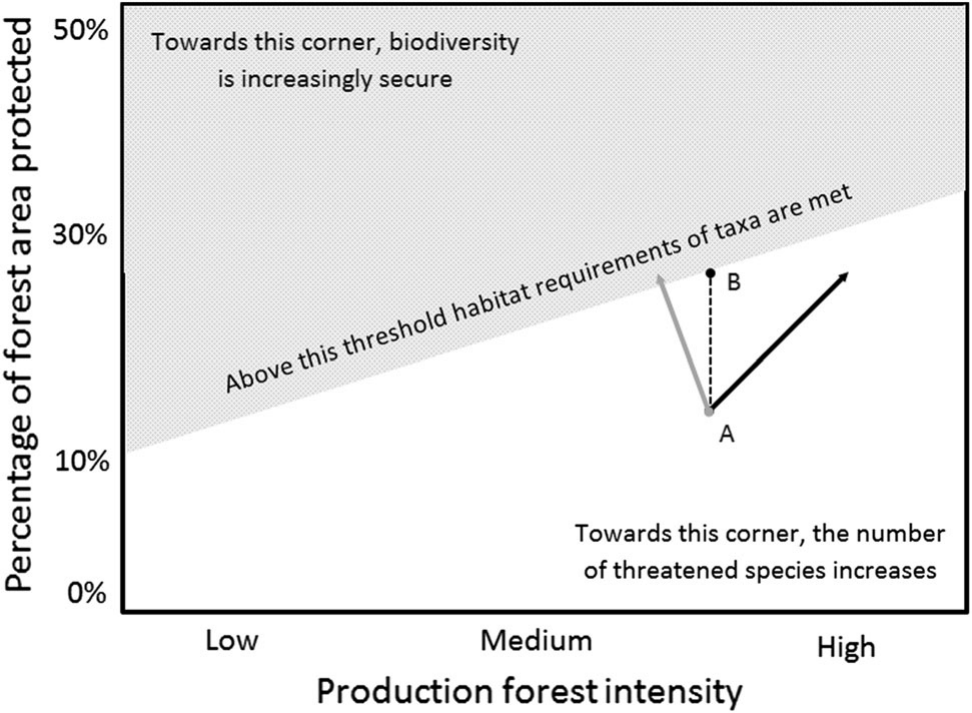
A conceptual framework illustrating the potential interdependence between protected forest areas and production forest intensity for forest habitat provision. We anchor the figure to estimates that 10–30% of productive forest lands requires protection to meet the species habitat requirements (Angelstam and Andersson 2001). The dashed line between ‘A’ and ‘B’ indicates the distance between a hypothetical current system state and meeting species’ threshold habitat requirements. The arrows indicate the two production forest trajectories considered (grey arrow = less intensive; black arrow = more intensive). Both arrows involve the same increase in protected forests (vertical distance on *y*-axis), but only the grey arrow meets species’ habitat requirements

Despite advancements to its multi-scale conservation efforts, the long-term viability of Sweden’s forest biodiversity has yet to be secured. We suggest that closing the remaining gap between the habitat requirements of forest species, and the habitat provided by Sweden’s forests, is more likely to occur if the multi-scale conservation approach is integrated with changes in the production forest matrix. To disregard this interdependence is to increase the risk that habitat gains in the protected aspects of the system are insufficient to compensate for habitat losses elsewhere (Fig. 2). Notably, this interdependence was acknowledged at the time of the 1994 Forestry Act, and helped justify forestry deregulation as a means to allow a greater diversity of management intensities (Lämås and Fries 1995; Bush 2010). Likewise, such interdependence was reflected in the first estimates of how much protected forest area was needed to maintain forest biodiversity in Sweden (9% in the north, 16% in the south), for which calculations depended in part on whether silvicultural practice could emulate natural forest conditions (Angelstam and Andersson 2001). However, whereas we are confident of this interdependence, large uncertainties remain regarding the precise nature of this relationship (Fig. 2). Deciphering this relationship is necessary to clarify how shortfalls in habitat provision by one aspect of the system (protected or production forest areas) may or may not be compensated by gains in another. To clarify this interdependence and related knowledge gaps (Table 1), we consider (below) two contrasting developmental trajectories for Sweden’s production forestry, and their respective implications for both habitat provision and the types of conservation tools needed. The extent to which these trajectories are taken will be shaped by a complex interplay of societal values, governance, path dependencies and practice (Table 2).

**Table 1:** The multi-scale conservation approach and production forest trajectories: Six key knowledge gaps and policy issues to be addressed in order to better integrate multi-scale conservation with production forest trajectories. The answers provided will be context and species specific

Key knowledge gaps and policy issues	
Key knowledge gaps Intensified forestry trajectory	What effect does forestry intensification have on the habitat contribution of production forest lands and the protected areas nested within?
	What type and extent of conservation action would adequately compensate for the habitat losses of a particular type and extent of forestry intensification?
Diversified forestry trajectory	When does the adoption of less intense forestry approaches allow for relaxed conservation requirements or protected area provision?
Policy issues	What costs and benefits are involved in the effective landscape-scale planning of forest natural resources and biodiversity conservation?
	What level of buffering should be baked into multi-scale conservation to compensate for unforeseen future forestry intensification?
	What threshold of forestry intensification should require compensatory conservation offsets beyond current requirements?

**Table 2:** A list of potential leverage points for diversifying forestry practice. Pathways are grouped according to different categories of leverage points, from deeper (paradigms) to shallower (practice) levers for instigating change in the forest system (see Abson et al. 2017). Deeper leverage points are more likely to be of international relevance. Where suitable, a selected reference is provided to further illustrate the pathway indicated

Categories of lever	Diversification opportunity and consideration	Diversification pathway
Intent (norms, values, goals and underlying paradigms)	Stewardship	Ensure that natural resource use balances the interests of society, future generations, private needs and other species (Worrell and Appleby 2000)
	Bio-perversity	Avoid bio-perversities, whereby negative biodiversity outcomes arise from a narrow focus on addressing a single environmental problem (Lindenmayer et al. 2012; Felton et al. 2016a)
	Bio-economy	Link the development of the bio-economy to a broad range of ecosystem services (Winkel 2017)
	Green infrastructure	Integrate green infrastructure with diversified silvicultural practice (Andersson et al. 2013)
Governance & Design (managing social structures and institutions)	Strengthen regulation	Link the intensity of silvicultural practice to the conservation actions required (see uncertainties Table 1)
	Landscape planning	Use it to enhance the means and efficiency by which biodiversity and production goals can be achieved (Michanek et al. 2018)
	Integrated forest and game management	Balance herbivore population densities and the availability of suitable forage to facilitate browsing sensitive silvicultural alternatives (Bergqvist et al. 2018)
	Forest Agency advisors	Ensure sufficient resources are provided to help balance production and biodiversity goals (Nordén et al. 2017)
	Forestry education	Ensure sufficient capacity among forest managers and advisors in alternative silvicultural practice
	Third party certification	A market-driven means of motivating the adoption of alternative silvicultural practice (FSC 2018)
	Ownership autonomy	Address forest owner reluctance to adopt alternative silvicultural prescriptions due to concerns that higher biodiversity will increase government control (Bjärstig and Kvastegård 2016; Bennich et al. 2018)
	Adaptive management & monitoring	Use different management objectives as an opportunity to test and resolve key uncertainties, and actively monitor the effects of new policies (Lindenmayer and Likens 2009; Rist et al. 2013)
Practice (system characteristics and feedbacks)	“An ounce of prevention is worth a pound of cure” -Benjamin Franklin	Prioritize the identification and protection of valuable habitats that still exist (Svensson et al. 2018).
	Build on forest owner diversity	Help the diversity of forest owner goals and ambitions match the forestry alternatives chosen (Ingemarson et al. 2006; Kindstrand et al. 2008; Eggers et al. 2014; KSLA 2017)
	Exploit natural regeneration opportunities	Seek win–win between reduced regeneration costs, natural regeneration, better aesthetics and diversified habitat provision (KSLA 2017; Lodin et al. 2017)
	Monitoring of forest parameters	Ensure forest metrics effectively capture the most relevant changes to forest habitat availability

### The intensification trajectory

In Sweden and other countries where the majority of forest land is used for wood production, the widespread adoption of more intensive forestry practices would decrease the diversity of forest habitats in those areas, and alter the effectiveness of conservation actions (Prugh et al. 2008; Franklin and Lindenmayer 2009). This is because the intensity of management in a matrix dominated by production forest can reduce the biodiversity benefits of reserves, set-asides and buffer zones (Aune et al. 2005; Johansson et al. 2018) via processes including habitat fragmentation and increasing edge effects on remnant forest patches (Haddad et al. 2015; Pfeifer et al. 2017; Nordén et al. 2018). A key uncertainty is thus how to design multi-scale conservation to keep pace with and compensate for reductions in habitat availability and connectivity that stem from different production forest intensification trajectories. The nature of this challenge is highlighted by the varied ways in which intensified forestry practices manifest (Box 1). Each of these practices has distinct impacts on the quality of available forest habitats, operate at varying spatial scales, and may be combined within a stand to compound net impacts.

The successive adoption of more intensive forestry practices could thereby limit the capacity of multi-scale conservation to meet species habitat requirements (Fig. 2). These habitat losses could potentially be ‘offset’ by linking forest management intensity to the extent and type of conservation action required. For example, habitat loss due to logging residue extraction could be mitigated by the creation of additional high stumps during harvest (Ranius et al. 2014). Similarly, the adoption of shorter rotation times could be offset by an increase in set-asides that compensate for the loss of mature forest conditions (Felton et al. 2017; Roberge et al. 2018). As the best choice of offset is unlikely to always occur within the area where intensification takes place, the adoption of landscape-scale planning (Tables 1, 2) would greatly improve the effectiveness of such integrated conservation efforts (Angelstam et al. 2011; Michanek et al. 2018). The advantage of spatial planning is that it allows the landscape-scale combination of distinct forest land-use categories, including protected and production forest lands, to better achieve both conservation and economic goals (Côté et al. 2010; Naumov et al. 2018). However, there are obstacles to implementing landscape-level management (Pawson et al. 2013), especially in regions, like southern Sweden, that are managed by hundreds of thousands of small-scale private forest owners (McDermott et al. 2010; Gustafsson et al. 2015). Furthermore, offsetting per se involves additional challenges (Maron et al. 2012), and reveals additional knowledge gaps (Table 1). For example, what threshold must be crossed for a new silvicultural practice to require additional compensatory conservation actions, and what conservation action is sufficient to adequately compensate for a specific intensified forestry practice? Not resolving these issues runs the risk that advances in multi-scale conservation (e.g. increases to protected forest areas) would not be sufficient to secure forest biodiversity if forestry intensifies (Fig. 2, black arrow).

### The diversification trajectory

What is potentially a more direct path to achieving biodiversity goals (Fig. 2, grey arrow) is to adopt a diversity of forest management alternatives (Box 2) that better overlap with the breadth of tree species and disturbance regimes found in Sweden’s natural and semi-natural forest systems (Fries et al. 1997; Angelstam 1998; Kuuluvainen 2009). If such diversified forestry practices are more widely adopted, this may correspondingly reduce the need for additional protected forest areas (leftward shift in Fig. 2). Diversified forestry approaches also provide a number of co-benefits. First, societal expectations increasingly favour managing production forests for a diverse range of goods and services (e.g. recreation, non-wood forest products; Gustafsson et al. 2012; Schwenk et al. 2012; Lindahl et al. 2017), generally requiring a range of silvicultural approaches (Van der Plas et al. 2016). Second, diversification is a recommended strategy for adapting forest lands to the uncertainties and altered disturbance regimes of climatic change (Pawson et al. 2013; Seidl et al. 2018). For example, the Swedish Forest Agency (SFA) recently concluded that the use of continuous cover forestry (CCF), broadleaf stands and mixed broadleaf production forests should increase (Bergquist et al. 2016). Their increased use would not only diversify forestry practice (SFA 2017, 2018b), and aid climate change adaptation (Felton et al. 2016a, c), but is also expected to improve the biodiversity and ecosystem services provided (Kuuluvainen et al. 2012b; Felton et al. 2016c; Hjältén et al. 2017; Joelsson et al. 2018). Finally, diversified forestry is also likely to increase the food supply for large browsing herbivores in the matrix, which may reduce browsing pressure on damage-sensitive young stands (Bergqvist et al. 2018).

**Box 2:** Three silvicultural means of increasing habitat availability as part of forestry diversification

*Mixed*-*species stands* Mixed-species stands involve the targeted production of two or more tree species, with limits on the extent one tree species can dominate the stand. Mixed-species stands are associated with higher biodiversity and the provision of a broader suite of ecosystem services (Felton et al. 2016c). In terms of habitat, mixtures increase the range of environmental conditions and resources provided, especially if the tree species grown are phylogenetically distinct and facilitate establishment by flora and fauna evolved to exploit either the mixture per se (Jansson and Andren 2003) or each tree species’ resources and structures (Jonsell et al. 1998). In Sweden, the increased use of broadleaf trees in Norway spruce stands benefits biodiversity, due in part to improved soil insolation and quality that favours vascular plants and associated taxa (Barbier et al. 2008). For example, adding birch to Norway spruce stands increases the diversity of birds, understory vegetation, saproxylic beetles and lichens (Felton et al. 2010; Lindbladh et al. 2017). Notably forest biodiversity in Sweden is also expected to benefit from increasing the use of broadleaf dominated production stands in general (Lindbladh et al. 2014; Felton et al. 2016b). *Uneven*-*aged management* In Fennoscandia, forest biodiversity can benefit from the increased use of uneven-aged management, due to its greater consistency with the finer spatio-temporal grains of natural disturbance regimes, and the associated habitat types provided (Kuuluvainen 2009; Kuuluvainen et al. 2012b). These benefits include increased horizontal and vertical structural heterogeneity, improved forest connectivity (Lindenmayer and Franklin 2002), and the more continuous provision of relatively mature trees and coarse woody debris (Atlegrim and Sjöberg 2004). By providing understory microclimate conditions associated with mature tree cover, uneven-aged management tends to favour species associated with later successional forest stages, including species of understory vascular plants, saproxylic beetles and other arthropods (Kuuluvainen et al. 2012b; Hjältén et al. 2017). Uneven-aged forestry can also be used to increase the multi-functional capacity of production forests (Peura et al. 2018). *Longer rotation times* Longer rotations generally reduce the ecological distinction between production and protected forest areas by better emulating natural disturbance regimes (Lindenmayer and Franklin 2002), and providing more of the microhabitats associated with older forests, including old or large trees, tree cavities, thick creviced bark and exposed wood (Siitonen 2012), deadwood of increased size and variety (Jonsson et al. 2006), and also favour dwarf shrubs (Hedwall et al. 2013). Furthermore, it increases opportunities for species colonization (Nordén et al. 2014). However, outcomes depend on thinning regimes (Roberge et al. 2016), and important microhabitats can occur in trees older than production goals allow (Ranius et al. 2009; Santaniello et al. 2017). Whereas longer rotations can also increase timber size, forest carbon storage, and improve water quality, shorter rotation times may instead be used to exploit increased growth rates and reduce disturbance risks (Roberge et al. 2016)	

### The need for leverage points

The degree to which intensive versus diversified forestry approaches are embraced over coming decades will depend on the extent that values, such as biodiversity conservation (Table 2), influence how the demand for raw material is met (Nilsson et al. 2011) and negative CO_2_ emissions achieved (Heck et al. 2018). It will also depend on how concepts like the ‘bio-economy’ (Pülzl et al. 2014) and ‘green infrastructure’ (Andersson et al. 2013; Snäll et al. 2016) are interpreted (Table 2). Strong production and economic incentives to pursue intensified approaches can also be expected to influence outcomes (Brukas and Weber 2009). For example, recent assessments suggest that the increased use of intensified silvicultural practices, including fertilization, exotic tree species and ditching, could provide Sweden with a 30% increase in production by the end of the century (SFA 2018a). Such production benefits can take precedence in forest management decisions, despite Sweden’s Forestry Act equating production and environmental goals (Ulmanen et al. 2012; Lindahl et al. 2017). The reasons for this are diverse, but stem in part from intensive forestry having received over 70 years of intellectual and economic investment, as well as extended periods of regulatory support (Lindkvist et al. 2012; Lindahl et al. 2017). Long-term investments have successfully increased the efficiency of intensive even-aged approaches via a supportive educational system (Blomström and Kokko 2003), technological developments (Nordansjö 2011), an industry tooled towards the processing of standard-sized conifer timber and pulpwood (SFA 2014; Södra 2018), and well-established and reliable markets for Norway spruce and Scots pine that stimulate further investment (Lindahl et al. 2017; Lodin et al. 2017). In addition, enhanced conifer seedlings that provide better growth, survival and economic returns are widely available (Jansson et al. 2017), further reinforcing the use of high-input regeneration methods.

If Sweden’s forest future is to involve more diversified forestry practices, this may require the identification of suitable ‘leverage points’ to instigate change (sensu Meadows 1999; Table 2). Leverage points are specified means of shifting social-ecological systems in a desired direction, for which ‘levers’ are classified as being ‘shallow’ (e.g. practical levers like taxes) or ‘deep’ (e.g. societal values), depending on their expected ability to instigate change (Abson et al. 2017; but see Manfredo et al. 2017). Multiple potential levers appear to be available in Sweden (Table 2), which may need to be exploited to achieve more diversified forestry. For example, approximately 30% of small-scale private forest owners have ‘conservation’ or ‘multiple’ objectives for their forests (Ingemarson et al. 2006), and multiple international studies have indicated the importance of such intrinsic values in motivating the adoption of conservation practices (Greiner and Gregg 2011; Mzoughi 2011). Ensuring that SFA advisors have the resources to reach such owners (Michanek et al. 2018) is a key lever for clarifying the potential benefits of silvicultural diversity including uneven-aged forestry, broadleaves and broadleaf mixtures (Bergquist et al. 2016, 2018). Without this capacity, industry-linked advisors can dominate this role (Andersson et al. 2017a), potentially overestimate the importance of production to some private forest owners (Kindstrand et al. 2008), and reinforce intensive forestry practices (Ulmanen et al. 2012; Nordén et al. 2017). An additional potential lever is to develop techniques for identifying those areas in which intensive regeneration approaches are likely to fail in production stands. If such sites can be determined beforehand, then the natural regeneration of broadleaves and conifers can be promoted for the benefit of both biodiversity and reduced establishment costs (KSLA 2017; Lodin et al. 2017). Likewise, ensuring that the population density of large herbivores (largely determined by hunting pressure) is balanced by the spatial and temporal distribution of suitably diverse forage will reduce the extent to which forest owners and managers are constrained by browsing damage concerns (Box 1) when choosing tree species for regeneration (Bergqvist et al. 2018). The FSC could also play an important role in diversification efforts, especially if the proposed national forest stewardship standard is adopted requiring an additional 5% of a property’s forest area be either set aside or managed using alternative practices like uneven-aged forestry (FSC 2018). This new requirement could aid the uptake of such alternatives by providing a financial motivation to forest owners to run trials, and likewise motivate forestry organizations working with advisory services to become more familiar with alternative silvicultural practices (Table 2; Stens et al. in press).

## Conclusion

We suggest that achieving forest biodiversity conservation goals in Sweden, and in similar contexts internationally, will largely be determined by how well multi-scale conservation is adapted to, and integrated with, ongoing changes in the production forest matrix. Specifically, the degree to which forestry intensification versus diversification trajectories are embraced will determine the types and extent of conservation measures needed to conserve forest biodiversity over coming decades. As long as the status of forest biodiversity remains insecure, and forest habitat remains quick to lose but slow to recover, the use of more intensive forestry should raise concerns regarding resultant habitat loss and fragmentation, and the continued effectiveness of past conservation strategies. In contrast, the use of diversified approaches should be easier to integrate with multi-scale conservation, and adds to the ‘tool-box’ of means by which biodiversity targets can be hit. Teasing out and addressing the associated knowledge gaps will be a complicated but essential part to charting the most feasible course towards securing the status of forest species. Furthermore, because the issues we raise involve social-ecological systems, finding suitable solutions to balancing production, climate change and biodiversity goals will demand insights from a wide range of academic disciplines and stakeholders. More generally, as the drivers of intensified forestry appear to be replicated wherever industrial-scale production forestry is practised (Puettmann et al. 2015), the need to resolve these issues likely extends to the many nations where production forests define the forest matrix, and protected forest areas are inadequate on their own to conserve forest biodiversity. For Sweden and other countries trying to protect their natural heritage under such circumstances, ensuring that the threshold habitat requirements of forest dependent species are met despite these complexities will be one of the key challenges of this century.
