# Correction to: Keeping pace with forestry: Multi-scale conservation in a changing production forest matrix

**DOI:** 10.1007/s13280-019-01291-x

**Published:** 2019-11-16

**Authors:** Adam Felton, Therese Löfroth, Per Angelstam, Lena Gustafsson, Joakim Hjältén, Annika M. Felton, Per Simonsson, Anders Dahlberg, Matts Lindbladh, Johan Svensson, Urban Nilsson, Isak Lodin, P. O. Hedwall, Anna Sténs, Tomas Lämås, Jörg Brunet, Christer Kalén, Bengt Kriström, Pelle Gemmel, Thomas Ranius

**Affiliations:** 1 Southern Swedish Forest Research Centre, SLU, Box 49, Rörsjöv 1, 230 53 Alnarp, Sweden; 2 Department of Wildlife, Fish, and Environmental Studies, Swedish University of Agricultural Sciences, 901 83 Umeå, Sweden; 3 Faculty of Forest Sciences, School for Forest Management, Swedish University of Agricultural Sciences, PO Box 43, 730 91 Skinnskatteberg, Sweden; 4 Department of Ecology, Swedish University of Agricultural Sciences, P.O. Box 7044, 750 07 Uppsala, Sweden; 5 Härnösand, Sweden; 6 Department of Forest Mycology and Plant Pathology, Swedish University of Agricultural Sciences, PO Box 7026, 750 07 Uppsala, Sweden; 7 Department of Historical, Philosophical and Religious Studies, Umeå University, 901 87 Umeå, Sweden; 8 Department of Forest Resource Management, Swedish University of Agricultural Sciences, 901 83 Umeå, Sweden; 9 National Forest Agency, Bryggargatan 19-21, 503 38 Borås, Sweden; 10 Department of Forest Economics, Swedish University of Agricultural Sciences, 90183 Umeå, Sweden

## Correction to: Ambio 10.1007/s13280-019-01248-0

In the original published article, the sentence “Nevertheless, semi-natural forest remnants continue to be harvested and fragmented (Svensson et al. 2018; Jonsson et al. 2019), and over 2000 forest-associated species (of 15 000 assessed) are listed as threatened on Sweden’s red-list, largely represented by macro-fungi, beetles, lichens and butterflies (Sandström 2015).”under the section **Introduction** was incorrect.

The correct version of the sentence is “Nevertheless, semi-natural forest remnants continue to be harvested and fragmented (Svensson et al. 2018; Jonsson et al. 2019), and approximately 2000 forest-associated species (of 15 000 assessed) are on Sweden’s red-list, largely represented by macro-fungi, beetles, lichens and butterflies (Sandström 2015).”

